# Prevalence of and Factors Associated With Adverse Maternal Obstetrical Events Among Teenage Mothers Delivering in a Tertiary Referral Hospital in Southwestern Uganda

**DOI:** 10.7759/cureus.66168

**Published:** 2024-08-05

**Authors:** Joseph Ngonzi, Wilson Birungi, Onesmus Byamukama, Arnold Kamugisha, Josephine Asiimwe, Moses Ntaro, Grace Nambozi, Leevan Tibaijuka, Charles Tushabomwe-Kazooba

**Affiliations:** 1 Obstetrics and Gynecology, Mbarara University of Science and Technology, Mbarara, UGA; 2 Business Administration, Mbarara University of Science and Technology, Mbarara, UGA; 3 Community Health, Mbarara University of Science and Technology, Mbarara, UGA; 4 Nursing, Mbarara University of Science and Technology, Mbarara, UGA; 5 Central Administration, Mbarara University of Science and Technology, Mbarara, UGA

**Keywords:** uganda, factors associated, adverse maternal outcomes, maternal child health, teenage pregnancy

## Abstract

Introduction: Many female teenagers in low-resource settings conceive, of which half are unplanned and end in many deaths in sub-Saharan Africa, accounting for the majority of the cases. Teenage pregnancy is associated sometimes with poor maternal, newborn, and child deaths.

Objectives: The aim of the study was to determine the prevalence, maternal obstetric outcomes, and factors associated with poor maternal obstetric outcomes among teenage mothers delivering at Mbarara Regional Referral Hospital.

Methods: This was a cross-sectional study carried out in a maternity ward at Mbarara Regional Referral Hospital, where 9,200 mothers deliver annually. All the women coming in for the delivery of their babies were consecutively approached for inclusion in the study. The women were enrolled in the post-delivery ward after delivery and interviewed with pretested questionnaires to capture the sociodemographic, obstetric, and medical profiles of the mothers. Factors were significant if the p*-*value was <0.05.

Results: Out of the 327 participants, the majority were rural dwellers (68.5%), married (75.8%), attained primary education (69.4%), had not used contraception (89%), and had had a planned pregnancy (63.3%). The prevalence of adverse maternal obstetrical events was 59.9%. The HIV-positive rate was 4.9%, and about half of the participants had delivered by cesarean section (41.6%). The participants' mean age was 18.4 years and SD 1.1. The mean number of antenatal care contacts attended was 4.59 and SD 1.9. The adverse maternal outcomes included episiotomy (30.9%), perineal tear (18.7%), premature rupture of membranes (10.1%), placenta abruption (5.2%), and pre-eclampsia/eclampsia (4%). Having a cesarean delivery was found to significantly reduce the occurrence of adverse maternal obstetric events among the participants by 97% (adjusted odds ratio (aOR) (95% CI) of 0.03 (0.02-0.06), p-value<0.001). Having a prior history of a miscarriage was significantly associated with the occurrence of adverse maternal obstetrical events among the participants (aOR (95% CI) of 6.55 (1.46-29.42), p-value0.014).

Conclusions: Slightly more than half of the teenage mothers had adverse maternal obstetrical outcomes, and a history of a miscarriage in previous pregnancies was significantly associated with adverse maternal obstetrical outcomes. Having a cesarean delivery was found to significantly reduce the occurrence of adverse maternal obstetric events among the participants. Teenage mothers are at a high risk of adverse maternal obstetrical outcomes, and close antepartum and intrapartum surveillance is recommended.

## Introduction

About 21 million girls aged 15-19 years in low-resource settings become pregnant, of which approximately 50% are unplanned and this results into approximately 12 million births worldwide [1,2]. The teenage age ranges between 10 and 19 years and is associated with a range of maternal and perinatal adverse effects [3-6]. Sub-Saharan Africa is responsible for about 50% of the global prevalence of teenage pregnancy and maternal, new-born, and child deaths [7,8].

Pregnancy in the teenage years is a high-risk condition and can cause major health and social challenges for the teenagers and society and about 24% of maternal mortality in Uganda occurs among teenagers and the inadequate access to optimal antenatal care (ANC) increases risks [9-11]. The adolescent age group is associated with adverse pregnancy outcomes [12,13]. Teenage pregnancies have been found to be associated with preterm premature rupture of membranes, gestational hypertension, pre-eclampsia, low Apgar scores, anemia, intrauterine growth restriction, and stillbirths [10,14,15]. Such a negative impact can affect the teenager in their entire life and carry over to the next generation [16]. In addition, operative vaginal deliveries, cesarean section rate, and low birth rate are significantly higher among teenagers compared to older ones [16].

Factors associated with teenage pregnancy are often categorized as sociodemographic, familial, cultural, and reproductive behavior [17]. Contributing factors to teenage pregnancies include non-urban dwellers, low education level, and inadequate communication on sexuality between the teenagers and their parents/guardians [18-22].

The aim of the study was to determine the prevalence of antepartum and intrapartum adverse maternal obstetric events and the associated factors among teenage mothers delivering at Mbarara Regional Referral Hospital.

## Materials and methods

Study design and setting

This was a cross-sectional study carried out in the maternity ward of Mbarara Regional Referral Hospital in southwestern Uganda. About 9,200 mothers deliver in the maternity ward and four out of 10 mothers undergo cesarean section while 15% of the mothers delivering at the facility are usually referred [23].

Inclusion and exclusion criteria

We included all the teenage mothers (10-19 years) in the postnatal ward at MRRH who delivered and consented to participate in the study. We excluded the teenage mothers who were incapacitated and their next-of-kin declined participation. 

Sampling of participants

All the women coming in for obstetric care were consecutively approached for inclusion in the study. The women were enrolled in the post-delivery ward after delivery. They were interviewed using pretested questionnaires.

Calculation of sample size

The sample size was calculated using OpenEpi, Version 3 using a proportion (28.6%) of teenage pregnancy taken from a study conducted in Ethiopia among female adolescents where a margin of error of 5% and 95% confidence interval were assumed [24]. The sample size calculated was 314 participants. We calculated a 10% non-response rate and obtained a final sample size of 349 participants.

Study procedure

A pretested questionnaire was administered to the mothers after informed consent (Appendix). Sociodemographic information such as maternal age, gravidity, religion, marital status, number of antenatal visits, employment status, and level of education was obtained. Information on maternal and fetal outcomes was also collected. The gestational age of pregnancy was derived from the last normal menstrual period. Occasionally we relied on the first-trimester ultrasound scan in patients who came for ANC early in pregnancy.

Definition of outcomes

The adverse maternal outcomes included the following: pre-eclampsia/eclampsia, premature rupture of membranes, placenta previa, placenta abruption, perineal tear, and episiotomy.

Ethical approval

We obtained ethical approval from the Mbarara University Institutional Review Board with approval number 09/05-17 and the Uganda National Council of Science and Technology (UNCST) with approval number HS967ES.

Data management and analysis

Descriptive statistics were calculated as numbers, percentages, and frequencies. Categorical outcome variables were compared by exposure using chi-square and Fisher’s exact tests. Multivariable backward stepwise analysis was performed using logistic regression, and only variables with a significance threshold of less than 0.2 were included in the final model. Measures of association were considered statistically significant at a p-value <0.05.

## Results

The number of participants who were recruited into the study was 327. Of the 327 participants in the study, the majority were rural dwellers (68.5%), married (75.8%), had attained primary education (69.4%), had a family member who had been pregnant before the age of 20 years (61.2%), had not used contraception (89%), and had had a planned pregnancy (63.3%). The prevalence of HIV was 4.9% and about half of the participants delivered by cesarean (41.6%). The mean age of the participants was 18.4 years and SD was 1.1 while the mean ANC attendance was 4.59 and SD was 1.9 as shown in Table 1.

**Table 1:** Sociodemographic, obstetric, and medical characteristics of teenage mothers delivering at Mbarara Hospital HIV, human immunodeficiency syndrome; ANC, antenatal care; SD, standard deviation.

Participant characteristics	n (%), N = 327	
Age in years (mean, SD)	18.41 (1.07)	
Residence type (n, %)		
Rural	224 (68.5)	
Urban	103 (31.5)	
Marital status (n, %)		
Single	70 (21.4)	
Separated/divorced	9 (2.8)	
Married	248 (75.8)	
Persons they live with (n, %)		
Husband	245 (74.9)	
Parent	43 (13.1)	
Relative/friend	33 (10.1)	
Alone	6 (1.8)	
How many pregnancies have you carried beyond seven months (n, %)?		
0	4 (1.2)	
1	291 (89.0)	
2	32 (9.8)	
Number of ANC attendances (mean, SD)	4.59 (1.93)	
Gestational age (mean, SD)	38.40 (2.62)	
Employed status (n, %)		
No	251 (76.8)	
Yes	76 (23.2)	
Employment sector (n, %)		
Formal	24 (31.6)	
Informal	52 (68.4)	
Level of education (n, %)		
No education	11 (3.4)	
Primary education	227 (69.4)	
Secondary education	89 (27.2)	
Currently attending school or in the last six months (n, %)		
No	316 (96.6)	
Yes	11 (3.4)	
Received sexuality education or counseling (n, %)		
No	156 (47.7)	
Yes	171 (52.3)	
Has anyone else in your family had pregnancy before 20 years (n, %)		
No	127 (38.8)	
Yes	200 (61.2)	
Used contraception before (n, %)		
No	291 (89.0)	
Yes	36 (11.0)	
Was this a planned pregnancy? (n, %)		
No	120 (36.7)	
Yes	207 (63.3)	
How do you feel about this pregnancy? (n, %)		
Happy	212 (64.8)	
Unhappy	86 (26.3)	
Indifferent	29 (8.9)	
HIV status (n, %)		
Negative	311 (95.1)	
Positive	16 (4.9)	
Ever had a miscarriage before? (n, %)		
No	298 (91.1)	
Yes	29 (8.9)	
Alcohol use (n, %)		
No	303 (92.7)	
Yes	24 (7.3)	
Delivery mode (n, %)		
Normal vaginal delivery	180 (55.0)	
Cesarean delivery	136 (41.6)	
Assisted vaginal delivery	11 (3.4)	

**Figure 1:**
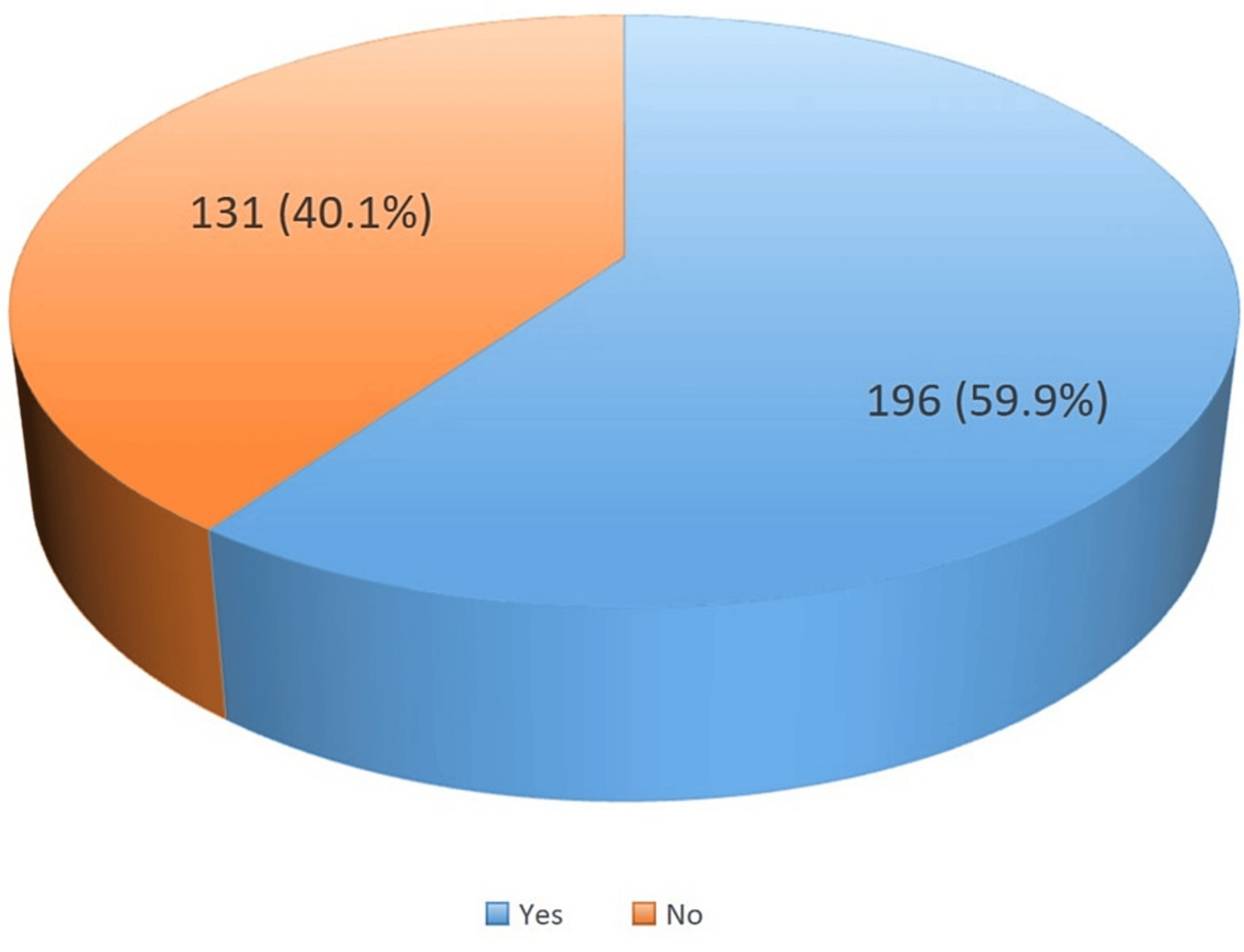
Prevalence of adverse obstetrical events among teenage girls

The prevalence of adverse maternal obstetrical events was 59.9%. The events studied included pre-eclampsia/eclampsia, premature rupture of membranes, placenta abruption, perineal tears, episiotomy, and vacuum extraction as shown in Figure 1.

Our study revealed that 30.9% had an episiotomy, 18.7% had a perineal tear, and 4% had pre-eclampsia/eclampsia as shown in Table 2. 

**Table 2:** Adverse maternal obstetrical events

Outcome	n (%), N = 327
Pre-eclampsia or eclampsia	
No	314 (96.0)
Yes	13 (4.0)
Premature rupture of membranes	
No	294 (89.9)
Yes	33 (10.1)
Placenta abruption	
No	310 (94.8)
Yes	17 (5.2)
Perineal tear	
No	266 (81.3)
Yes	61 (18.7)
Episiotomy	
No	226 (69.1)
Yes	101 (30.9)

There were significantly more adverse maternal obstetrical events among participants delivered by cesarean compared to vaginal delivery (adjusted odds ratio (aOR) (95% CI) of 0.03 (0.02-0.06), p-value <0.001). Having a prior history of a miscarriage was significantly associated with the occurrence of adverse maternal obstetrical events among the participants (aOR (95% CI) of 6.55 (1.46-29.42), p-value 0.014) (Table 3).

**Table 3:** Binary logistic regression to identify factors associated with adverse maternal events among teenage pregnancies cOR, crude odds ratio; aOR, adjusted odds ratio; HIV, human immunodeficiency virus; ANC, antenatal care; SD, standard deviation.

Characteristics	Univariate		Multivariable	
	cOR (95% CI)	p-Value	aOR (95% CI)	p-Value
Age in years	0.10 (0.81, 1.23)	0.973	1.15 (0.82, 1.61)	0.433
Urban residence type	1.46 (0.90,2.37)	0.129	1.68 (0.83, 3.39)	0.151
Marital status				
Separated/Divorced	4.44 (0.53, 37.61)	0.249	8.99 (0.80, 101.22)	0.076
Married	0.76 (0.44, 1.31)	0.320	0.97 (0.40, 2.36)	0.954
Been pregnant 2 or more times	1.06 (0.59, 1.88)	0.852	0.59 (0.19, 1.82)	0.360
Number of ANC attendances	0.92 (0.82, 1.03)	0.152	0.90 (0.74, 1.09)	0.280
Employed	1.28 (0.75, 2.19)	0.358	1.11 (0.50, 2.44)	0.800
Education level				
Primary	1.18 (0.35, 3.98)	0.791	1.35 (0.24, 7.62)	0.733
Secondary	1.48 (0.42, 5.25)	0.540	0.97 (0.18, 5.94)	0.970
Ever used contraception				
Yes	0.64 (0.32, 1.27)	0.200	0.34 (0.11, 1.10)	0.072
HIV status				
Positive	0.286 (0.10, 0.84)	0.023	1.54 (0.38, 6.29)	0.544
History of miscarriage				
Yes	1.85 (0.79, 4.30)	0.156	6.55 (1.46, 29.42)	0.014
Alcohol use				
Yes	0.31 (0.13, 0.74)	0.008	0.33 (0.10, 1.13)	0.077
Mode of delivery				
Cesarean	0.04 (0.02, 0.08)	<0.001	0.03 (0.02, 0.06)	<0.001

## Discussion

The study was done to determine the prevalence of antepartum and intrapartum adverse maternal obstetric events and the associated factors among teenage mothers delivering at Mbarara Regional Referral Hospital. In our study, the majority of the participants were rural dwellers, married, had attained only primary education, and had not used contraception. The prevalence of adverse maternal obstetrical events was 59.9%. About half of the mothers had delivered by cesarean (41.6%), had an episiotomy (30.9%), had a perineal tear (18.7%), and had pre-eclampsia/eclampsia (4%). There were significantly more adverse maternal obstetrical events among the participants who delivered by cesarean compared to vaginal delivery (aOR (95% CI) of 0.03 (0.02-0.06), p-value <0.001). Having a prior history of a miscarriage was significantly associated with the occurrence of adverse maternal obstetrical events among the participants (aOR (95% CI) of 6.55 (1.46-29.42), p-value 0.014).

The adverse obstetrical maternal events are similar to the findings from other studies where teenage and adolescent pregnancies have been found to be associated with higher risks of adverse pregnancy outcomes such as pre-eclampsia/eclampsia, premature rupture of membranes, placenta abruption, and perineal tears [2,23-26]. The possible explanation for conditions such as pre-eclampsia can be correlated to a lack of a regular ovulatory menstrual cycle, which can cause defective decidualization, leading to faulty placentation with a resultant remodeling of spiral arteries, eventually leading to pre-eclampsia [27]. Teenagers are more prone to PROM because they are more prone to underdiagnosed or diagnosed infections leading to PROM. The underdiagnosed infections increase inflammatory markers such as interleukins and prostaglandins, resulting in chorioamniotic and decidual inflammation [28].

The finding of the history of a miscarriage being associated with adverse maternal obstetrical events is similar to the results from other studies where the history of recurrent miscarriage was found to be associated with adverse maternal outcomes [29,30]. Teenagers who become pregnant demonstrate a greater risk for substance use, including alcohol and cigarettes, and there is a resultant effect on the perinatal outcomes [31]. The rates of sexually transmitted diseases (STDs) such as chlamydia, gonorrhea, and primary and secondary syphilis are increasing among teenagers and young women [32]. This may be due to hormonal changes occurring in adolescent girls that cause cervical ectopy (presence of columnar cells on the outer surface of the cervix), which are more susceptible to STIs [33]. STDs like syphilis are associated with adverse perinatal events such as stillbirths, miscarriages, and newborn deaths and chromosomal aberrations like structural alteration or abnormal chromosomal numbers [32,33]. Without treatment, more than 40% of syphilis-infected pregnancies may result in miscarriage, stillbirth, or neonatal death [34]. Previous studies have shown that women <19 years at their first pregnancy have a 37% heightened risk of having an abortion in their lifetime [35,36]. A potential explanation is that adolescent women may feel constrained by strict parental control, fostering a fear of parental rejection. Additionally, the lack of financial resources, combined with the fear of being unable to provide for a newborn baby, could drive them to consider unsafe abortions [35,37,38]. Lack of good nutritional status and a lack of partner support [39] could be other reasons that lead to spontaneous abortions.

Having a cesarean delivery was found to significantly reduce the occurrence of adverse maternal obstetrical events among the participants by 97% (aOR (95% CI) of 0.03 (0.02-0.06), p-value <0.001). The incomplete development of the maternal pelvis could determine an inability of the birth canal to allow the passage of the fetus and cause an increase in operative deliveries [40,41]. Cesarean delivery in our study was however protective of the adverse maternal outcomes because cesarean section can help optimize fetal outcomes when conducted timely before fetal compromise sets in [42]. The adverse associations of the cesarean section such as maternal sepsis, obstetrical hemorrhage, and organ damage have been reduced to the extent that cesarean section is as safe as vaginal delivery [43].

In our study, the majority were rural dwellers, married, attained only primary education, and had not used contraception. Consistent with the results from previous studies [44], teenage mothers predominantly reside in rural areas and this suggests a strong correlation between adolescent pregnancy and social factors. Given that adolescent pregnancy is more prevalent in socially deprived societies, these social factors can influence the adequacy of prenatal care among teenagers, potentially resulting in a higher prevalence of poor pregnancy outcomes among economically disadvantaged adolescents [45]. The majority of the teenagers in our study had not used contraception, and because of psychological immaturity, teenagers often fail to grasp the importance of family planning, leading them to engage in risky sexual behavior and become pregnant while still attending school and residing with their parents [46]. Our study reveals that a majority of teenage mothers are significantly more likely to have a low level of education levels, consistent with the findings from studies in other nations [46-48]. Teenage girls frequently discontinue their education due to pregnancy or childbirth, with issues at school and academic underperformance sometimes preceding pregnancy; for some teenage girls experiencing academic difficulties, motherhood may seem appealing, but when these factors converge, young mothers encounter diminished career prospects, often leading to reduced lifetime earnings [46,48]. 

Strengths

There are a few studies that have looked at adverse maternal obstetrical outcomes among teenagers in sub-Saharan Africa. Our study highlights the need to objectively optimize antepartum and intrapartum monitoring among teenage mothers.

Limitations

Adverse maternal obstetrical outcomes also occur in the postpartum period. These include postpartum hemorrhage, postpartum infections, and obstetrical fistula. By design of our study, the postpartum adverse outcomes were not measured, yet this would have given us a more comprehensive spectrum of adverse outcomes.

## Conclusions

The prevalence of adverse maternal obstetrical outcomes among teenage mothers at Mbarara Hospital is high and a history of a miscarriage in the previous pregnancies was significantly associated with adverse maternal obstetrical outcomes. There were significantly more adverse maternal obstetrical events among participants who delivered by cesarean compared to vaginal delivery. Teenage mothers are at a high risk of adverse maternal obstetrical outcomes, and close antepartum and intrapartum surveillance is recommended. Intrapartum monitoring can be optimized by the provision of monitoring equipment such as cardiotocogram machines and training healthcare providers on the use and interpretation of CTGs. Antepartum monitoring can be achieved by optimizing the ANC for these mothers. There is a need to study the interventions to reduce teenage pregnancies in our setting.

## Appendices

**Table 4:** Questionnaire for teenage pregnancy: Experiences and perinatal and obstetric outcomes at a tertiary referral hospital in southwestern Uganda

S. No	Question	Response
Sociodemographic characteristics		
	How old are you? State the age in years	
	What is your religion?	Catholic
		Protestant
		Pentecostal
		Muslim
		e. Other (such as Seventh Day Adventist, Jehovah Witness, traditional religion, atheist, etc.)
	Where do you reside?	Rural
		Urban Name:
	What is your marital status?	Single
		Separated/Divorced
		Married
	Who do you live with?	Husband
		Parent
		Relative/friend
		Alone
	How many times have you been pregnant?	1
		2-4
		5 or more
	How many pregnancies have you carried beyond 7 months?	
	How many pregnancies have you carried below 7 months?	
	How many times have you attended ANC?	
	What was the first day of the last normal menstrual period (if not known, calculate GA from first trimester ultrasound)	Calculate and record gestational age
	Are you employed?	Yes
		No
	If employed, what sector?	Formal
		Informal
	What is your level of education?	No education
		Primary education
		Secondary education
		More than secondary education
	Are you currently attending school or have you attended in the last six months?	Yes
		No
	About sexual education: Have you ever received guidance or counselling from your parents or aunty or other relatives about sex?	Yes
		No
	Has anyone else in your family had pregnancy before 20 years?	Yes
		No
Reproductive behavior		
	Age at first sexual intercourse	
	Have you used contraception before?	Yes
		No
	If yes to 18 above, which type of contraception did you use?	Implants (implanon, Jadelle)
		Injectaplan
		IUCD
		Oral contraceptives
		Others
	For how long did you use the contraception?	State in months
	Was this pregnancy planned?	
	What was the reason you conceived? (if yes in 20 above)	
	If no to 20 above, under what circumstances did you conceive?	Transactional sex
		Sexual violence
		Adolescent experimentation
		Others: …………………….
	How do you feel about this pregnancy?	Happy
		Unhappy
		Indifferent
	What is your HIV status?	Negative
		Positive
		Unknown
	If positive, are you on ARVs?	Yes
		No
	Have your ever miscarried before?	Yes No
	What type of miscarriage?	Spontaneous
		Induced
	Concerning an induced abortion, was the decision taken by yourself or you were influenced or advised by a peer or another person?	Yes
		No
	If yes to 26 above, who influenced or advised you to induce the miscarriage?	State here
Sociocultural behavior		
	Have you been influenced negatively by your friends toward sexual acts?	Yes
		No
	Has an older man (above 25) ever pushed you to have sex with him	Forced/coerced
		Induced with money or presents
		Threatened if no sex
		Consensual
	Do you drink alcohol?	Yes
		No
	If yes, how much alcohol?	A little
		Excess and get drunk
		At least 3 times a week
Maternal outcomes: Were any of the following outcomes present or absent?		Record ‘Y’ for present ‘N’ for absent
	Preeclampsia or eclampsia?	
	Premature rupture of membranes?	
	Placenta abruption?	
	Vaginal delivery?	
	Perineal tear?	
	Episiotomy?	
	Cesarean section?	
	Assisted vaginal delivery, e.g vacuum extraction	
Fetal/Neonatal outcomes		
	What was the gestational age at birth (Complete weeks of gestation)	<37-42
		≥42
	Did you have a miscarriage?	
	Birth weight (kg)	<2.5
		≥2.5-4
		>4
	Apgar score at 5 minutes	<7
		>7
	Was there a birth defect?	
	Still birth	
	Neonatal death	
Experience		
	In your words, explain how you felt when you had your initial sexual contact?	
	What circumstances put you in position to have sex that led to pregnancy and delivery?	
	Explain to me how you felt after discovering that you were pregnant?	
	In your words, tell me how you went through the pregnancy period	
	Are there actions that you did after discovering that you are pregnant? These could have been to protect you or even put yourself in harm	
	Tell me if you got any bad effects that still affect you after the pregnancy. These could have arisen while pregnant, during delivery, or after the pregnancy	
